# Congenital pulmonary airway malformations: prenatal markers, perinatal outcomes, and long-term follow-up experience

**DOI:** 10.1590/1806-9282.20260047

**Published:** 2026-07-31

**Authors:** Gökhan Ünver, Sercan Serin, Hüseyin Ekici, Handan Çelik, Miğraci Tosun

**Affiliations:** 1 Ondokuz Mayıs University, Faculty of Medicine, Department of Obstetrics and Gynecology, Perinatology Unit – Samsun, Türkiye.

**Keywords:** Prenatal diagnosis, Spontaneous regression, Perinatal care, Respiratory system abnormalities

## Abstract

**OBJECTIVE:**

The aim of this study was to evaluate prenatal characteristics, associated anomalies, and perinatal and postnatal outcomes of fetuses diagnosed with congenital pulmonary airway malformation at a tertiary referral center.

**METHODS:**

This retrospective study analyzed data from 56 fetuses diagnosed prenatally with congenital pulmonary airway malformation by ultrasonography between January 2015 and December 2024. Maternal age, gestational age at diagnosis and delivery, lesion localization and ultrasonographic type, congenital pulmonary airway malformation volume ratio, presence of hydrops fetalis and associated anomalies, karyotype results, perinatal losses, postnatal surgical requirement, and long-term outcomes were evaluated.

**RESULTS:**

The median gestational age at diagnosis was 23 weeks (range: 15–32). Lesions were located in the right lung in 58.9% and in the left lung in 41.1% of cases. Mixed lesions were most common (44.6%), followed by microcystic (30.4%) and macrocystic (25.0%) types. Congenital pulmonary airway malformation volume ratio >1.6 was observed in 17.9% of cases, and hydrops fetalis developed in 12.5%. Associated structural anomalies were detected in 12.5% of fetuses. Pregnancy termination, intrauterine fetal demise, and neonatal death occurred in 7.1, 1.8, and 1.8% of cases, respectively. Spontaneous lesion regression was observed in 62.5% of cases, while only 12% of live-born infants required postnatal surgery. During a mean follow-up of 72.5 months (range: 22–96), no congenital pulmonary airway malformation-related mortality was observed.

**CONCLUSION:**

Most fetuses with prenatal congenital pulmonary airway malformation diagnosis have favorable perinatal and long-term outcomes. High congenital pulmonary airway malformation volume ratio and hydrops fetalis are key predictors of adverse outcomes, whereas conservative management is safe and effective in asymptomatic cases.

## INTRODUCTION

Congenital pulmonary airway malformation (CPAM) is the most frequently diagnosed congenital lung lesion in the neonatal period, with an estimated incidence of approximately 1 in 25,000–35,000 live births^1^. Its pathogenesis is thought to involve a disruption in normal lung development during early gestation (between the 7th and 15th weeks). Abnormal proliferation of terminal bronchioles and suppression of normal alveolar development result in hamartomatous lesions that are supplied by pulmonary arteries and connected to the bronchial tree^2,3^.

The clinical course of CPAM is highly variable. While some lesions remain stable or demonstrate spontaneous regression during the prenatal period, others may show progressive growth, leading to complications such as polyhydramnios, cardiovascular compression, fetal hydrops, and, rarely, intrauterine fetal demise. Postnatally, patients may present with respiratory distress or remain completely asymptomatic. The optimal management of asymptomatic cases remains controversial, particularly regarding the choice between conservative follow-up and prophylactic surgical resection^4^.

In this study, we aimed to retrospectively evaluate the demographic characteristics, prenatal findings, perinatal and postnatal outcomes, and clinical management strategies of fetuses diagnosed prenatally with CPAM at a tertiary referral center.

## METHODS

All pregnancies diagnosed prenatally with CPAM by ultrasonography between January 2015 and December 2024 were included in the study. A total of 56 fetuses diagnosed during the study period were analyzed.

Maternal age, gestational age at diagnosis, lesion localization (right or left lung), ultrasonographic lesion type, presence of associated structural or chromosomal anomalies, karyotype analysis results, and fetal magnetic resonance imaging (MRI) findings were obtained from hospital medical records.

Perinatal and postnatal outcomes, including gestational age at delivery, birth weight, fetal sex, pregnancy termination, intrauterine or neonatal loss, spontaneous lesion regression, postnatal surgical requirement, and duration of long-term follow-up, were recorded. After data collection, all families were contacted by telephone to obtain updated information regarding the current clinical status of the patients.

**Figure 1:**
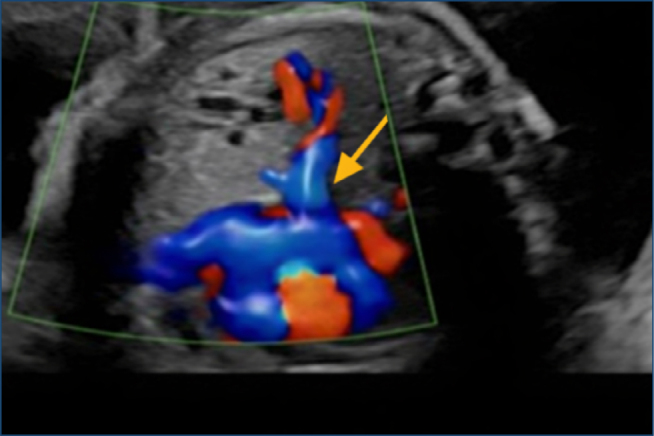
Color Doppler imaging demonstrates pulmonary arterial branching within the lesion.

All fetal ultrasonographic examinations were performed by experienced perinatology specialists. Lesion volume was calculated using the ellipsoid formula (length×width×anteroposterior diameter×0.52). The CPAM volume ratio (CVR) was obtained by dividing the calculated lesion volume by the fetal head circumference. CVR measurements were performed at the time of diagnosis and during weekly or biweekly follow-up assessments, and the highest recorded CVR value was used for prognostic evaluation. Fetuses with a CVR ≥1.6 were classified as high risk^5^. Lesions were categorized according to ultrasonographic appearance as macrocystic (single or multiple large cysts), microcystic (solid, hyperechogenic lesions), or mixed type. Color and power Doppler ultrasonography was used to demonstrate pulmonary arterial branching within the lesion, supporting the diagnosis of CPAM (Figure 1). Fetal MRI was utilized in selected cases for differential diagnosis or when additional anomalies were suspected.

Statistical analysis was performed using SPSS software (Statistical Package for the Social Sciences), version 25.0. The normality of continuous variables was assessed using the Kolmogorov-Smirnov test. Normally distributed variables were presented as mean±standard deviation (SD), whereas non-normally distributed variables were expressed as median (minimum–maximum). Categorical variables were reported as numbers and percentages, n (%).

## RESULTS

A total of 56 fetuses prenatally diagnosed with CPAM were included in the study. The mean maternal age was 26.5±4.2 years (range: 18–39). The median gestational age at diagnosis was 23 weeks (range: 15–32), while the median gestational age at delivery among live-born infants was 38 weeks (range: 37–39). The mean birth weight of live-born infants was 3,226±447 grams. The demographic and baseline perinatal characteristics are summarized in Table 1.

**Table 1:** Demographic and baseline perinatal characteristics of the study cohort (n=56).

Variable	n (%) or mean±SD/median (range)
Maternal characteristics	
Maternal age (years)	26.5±4.2
Fetal/neonatal characteristics	
Gestational age at diagnosis (weeks)	23 (15–32)
Gestational age at delivery (weeks)	38 (37–39)
Birth weight (grams)	3,226±447
Gender (male/female)	31 (55.4%)/25 (44.6%)
Delivery and perinatal outcomes	
Mode of delivery (vaginal/cesarean)	12 (23.5%)/39 (76.5%)
Term delivery (≥37 weeks)	44 (86.3%)
Preterm delivery (<37 weeks)	7 (13.7%)
Overall pregnancy outcome	
Live birth	51 (91.1%)
Survival to discharge	50 (89.3%)
Pregnancy termination	4 (7.1%)
Intrauterine fetal demise	1 (1.8%)
Neonatal death	1 (1.8%)

SD: standard deviation.

Of the fetuses, 55.4% were male (n=31) and 44.6% were female (n=25). Lesions were located in the right lung in 58.9% (n=33) and in the left lung in 41.1% (n=23) of cases. According to ultrasonographic classification, 44.6% of lesions were mixed type, 30.4% were microcystic, and 25.0% were macrocystic.

Polyhydramnios was detected in 14.3% (n=8) of cases, and mediastinal shift was observed in 17.9% (n=10). A CVR ≥1.6 was present in 17.9% (n=10) of fetuses. Hydrops fetalis developed in 12.5% (n=7) of cases, all of which were associated with CVR ≥1.6 and mediastinal shift. Importantly, mediastinal shift was not observed as an isolated finding in our cohort and was consistently accompanied by other markers of disease severity, such as increased CVR and/or hydrops. The cardiomediastinal shift angle was not routinely calculated as part of our standard clinical protocol. Overall, these findings were predominantly observed in fetuses with macrocystic or mixed lesions.

Associated structural anomalies were identified in 12.5% (n=7) of fetuses. Major anomalies were detected in three cases (pulmonary atresia with intact ventricular septum, muscular ventricular septal defect, and unilateral multicystic dysplastic kidney), while minor anomalies were found in four cases (three cases of renal pelvis dilatation and one aberrant right subclavian artery). Karyotype analysis was performed in 20 cases (35.7%), all of which yielded normal results. Fetal MRI was performed in 14 cases (25.0%) for prenatal differential diagnosis and did not provide additional diagnostic information beyond ultrasonography. The prenatal ultrasonographic findings and antenatal management are presented in Table 2.

**Table 2:** Prenatal ultrasonographic findings and antenatal management (n=56).

Prenatal variable	n (%)
Lesion characteristics	
Lesion location (right/left lung)	33 (58.9%)/23 (41.1%)
Ultrasonographic type	
Mixed	25 (44.6%)
Macrocystic	14 (25.0%)
Microcystic	17 (30.4%)
High-risk markers	
CVR ≥1.6	10 (17.9%)
Mediastinal shift	10 (17.9%)
Polyhydramnios	8 (14.3%)
Fetal hydrops	7 (12.5%)
Associated anomalies	7 (12.5%)
Major anomalies^*^	3 (5.4%)
Minor anomalies^**^	4 (7.1%)
Cases undergoing karyotype analysis, n (%)	20 (35.7%)
• Abnormal karyotype	0 (0%)
Antenatal corticosteroid administration	2 (3.6%)
Gestational age at steroid administration (weeks)	Median 27 weeks (range: 26–28)
Fetal MRI performed	14 (25.0%)

*Major anomalies: pulmonary atresia with intact ventricular septum (n=1), muscular ventricular septal defect (n=1), and unilateral multicystic dysplastic kidney (n=1).

**Minor anomalies: Renal pelvis dilatation (n=3), aberrant right subclavian artery (n=1). CVR: CPAM volume ratio; MRI: magnetic resonance imaging.

Notably, four cases (7.1%) complicated by hydrops fetalis resulted in pregnancy termination before 20 weeks of gestation, while one case (1.8%) was lost due to intrauterine fetal demise at 28 weeks of gestation. However, in two selected cases with macrocystic lesions and a CVR greater than 1.6, antenatal betamethasone was administered between 28 and 30 weeks of gestation due to elevated CVR (≥1.6), mediastinal shift, and early signs of hydrops, indicating increased risk of clinical deterioration. A standard regimen of betamethasone (two doses of 12 mg administered intramuscularly 24 h apart) was used. Despite treatment, no regression or reduction in lesion size was observed. In both cases, emergency cesarean delivery was required at 37 weeks of gestation following the development of pericardial effusion and ascites. Both of these infants underwent surgical intervention within the first week postpartum. In one case (1.8%), neonatal death occurred on postnatal day 10 due to an associated diagnosis of pulmonary atresia with intact ventricular septum. Among live-born infants, 12 (23.5%) were delivered vaginally, while 39 (76.5%) were delivered by cesarean section. The high cesarean section rate was primarily attributable to a history of previous cesarean delivery in a substantial proportion of the mothers, rather than CPAM-related fetal indications.

Among live-born infants who were followed postnatally, spontaneous regression of the lesion was observed in 62.5% (n=35) either during the prenatal or postnatal period. Regression was confirmed by postnatal lung ultrasonography or chest radiography. Postpartum imaging data were unavailable for the remaining 15 cases; however, telephone interviews with the families revealed that pediatric follow-up evaluations showed no additional clinical problems in these infants.

The rate of postnatal surgical intervention was 12% (n=6). Among surgically treated cases, three lesions were macrocystic (CPAM type 1), one was mixed (CPAM type 2), and two presented as predominantly microcystic lesions, including one case ultimately diagnosed as pulmonary sequestration on pathological examination. Half of the surgically treated infants (n=3) required early surgery within the first 7 days of life due to respiratory distress, and all of these cases had a CVR ≥1.6. The remaining three infants underwent surgery at 5–6 months of age due to recurrent respiratory tract infections, frequent hospitalizations, and lack of lesion regression. In one case, despite a CVR <1.6, a dominant macrocystic component and symptomatic clinical course constituted the indication for surgery. Among surgically treated neonates, the median length of neonatal intensive care unit stay was 12 days (range: 6–29), and the median total hospital stay was 14 days (range: 6–29).

Pathological examination confirmed the diagnosis of CPAM in five of the six surgically treated cases. In one case, although prenatal ultrasonography and fetal MRI findings were suggestive of a microcystic CPAM, the final pathological diagnosis was bronchopulmonary sequestration. Accordingly, the concordance rate between prenatal diagnosis and postnatal pathological findings among surgically treated cases was 83.3% (5/6). During a mean postnatal follow-up period of 72.5 months (range: 22–96), no CPAM-related mortality or malignancy was observed. Postnatal management, surgical outcomes, and long-term follow-up are detailed in Table 3.

**Table 3:** Postnatal management, surgical outcomes, and longterm follow-up.

Outcome variable	n (%) or median (range)
Clinical course	
Spontaneous lesion regression	35 (62.5%)
Need for surgical intervention	6 (12.0%)
Surgical details (n=6)	
Indication for surgery	
Respiratory distress	3 (50%)
Progressive lesion growth/recurrent infections	3 (50%)
Timing of surgery	
Early (postnatal days 2–10)	3 (50%)
Late (3–5 months)	3 (50%)
Hospitalization (surgical cases only)	
Neonatal intensive care unit stay (days)	12 (6–29)
Total hospital stay (days)	14 (6–29)
Postoperative complications	
Surgical site infection	1 (16.7%)
Pathological findings	6
CPAM type 1	3 (50%)
CPAM type 2	1 (16.7%)
CPAM type 3	1 (16.7%)
Bronchopulmonary sequestration	1 (16.7%)
Concordance between prenatal and postnatal pathological diagnosis	5/6 (83.3)
Long-term follow-up	
Postnatal follow-up duration (months)	72.5 (22–96)
Survival rate	100%

CPAM: congenital pulmonary airway malformation.

## DISCUSSION

With the widespread use of antenatal ultrasonography and improvements in imaging quality, most CPAM cases are now diagnosed during the intrauterine period. Previous studies have reported that CPAM is most commonly diagnosed between 18 and 34 weeks of gestation^6^. The median gestational age at diagnosis of 23 weeks in our cohort is consistent with these reports.

In addition to its prognostic role, CVR appears to reflect the underlying pathophysiological cascade associated with lesion growth. In our cohort, fetuses with a CVR ≥1.6 showed significantly higher rates of mediastinal shift and pleural/abdominal effusion compared with those with CVR <1.6, along with a trend toward increased amniotic fluid depth. These findings support the concept that progressive lesion enlargement leads to mass effect, resulting in mediastinal compression, impaired venous return, reduced fetal swallowing, and subsequent development of effusions and polyhydramnios^7,8^. From a clinical perspective, close surveillance of mediastinal shift, effusions, and amniotic fluid changes in fetuses with CVR ≥1.6 is essential to anticipate clinical deterioration and allow timely intervention. Recent studies have suggested that objective assessment of mediastinal displacement using the cardiomediastinal shift angle (CMSA) may improve risk stratification in CPAM. Increased CMSA has been associated with adverse perinatal outcomes and the development of hydrops, supporting mediastinal shift as a marker of disease severity rather than an isolated finding. Although CMSA was not routinely measured in our cohort, our observations are consistent with these findings, as mediastinal shift was invariably accompanied by elevated CVR and other signs of severe disease^9^.

Prenatal ultrasonography remains the cornerstone of CPAM diagnosis and management. In our study, fetal MRI did not provide additional diagnostic value beyond ultrasonography in 14 cases. Moreover, in one case, followed prenatally as CPAM based on both ultrasonographic and MRI findings, postnatal pathological evaluation revealed bronchopulmonary sequestration. This finding highlights that even fetal MRI may have limitations in achieving definitive diagnostic accuracy in the differential diagnosis of congenital lung lesions. Therefore, fetal MRI should likely be reserved for selected cases in which differential diagnosis is particularly challenging or when complex associated anomalies are suspected, as its routine use may reduce cost-effectiveness and lead to unnecessary procedures^10^.

One of the most important components of prenatal CPAM evaluation is the exclusion of associated structural and chromosomal anomalies. Although chromosomal abnormalities are reported to be rare in association with CPAM, genetic testing is recommended in the presence of additional structural anomalies^11^. In our cohort, all cases undergoing karyotype analysis had normal results, supporting the concept that CPAM is predominantly an isolated pathology.

Antenatal corticosteroid therapy has been proposed for high-risk CPAM, particularly in fetuses with elevated CVR or evolving hydrops. Fetal intervention may be considered at any CVR value in the presence of hydrops, rapid lesion growth, or polyhydramnios. Maternal corticosteroids appear to be more effective in predominantly microcystic lesions, whereas macrocystic CPAMs are more often managed with invasive approaches such as thoracoamniotic shunting^12^. Corticosteroids are currently considered the first-line therapy for fetuses with hydrops or for those deemed at high risk of developing hydrops based on a CVR greater than 1.6. Nevertheless, there remains no clear consensus regarding the optimal timing and frequency of antenatal corticosteroid administration^13^.

As reported in the literature, macrocystic CPAMs with low CVR values and without mediastinal compression may remain stable or regress during later stages of pregnancy. This observation supports conservative management regardless of lesion morphology in asymptomatic, low-risk cases^12,13^.

In our cohort, surgically treated cases were predominantly characterized by high-risk prenatal features, including a CVR ≥1.6, mediastinal shift, and hydrops fetalis. In contrast, cases that demonstrated spontaneous regression were consistently associated with low-risk prenatal characteristics and remained asymptomatic during the neonatal period. In our cohort, macrocystic morphology was more common among surgically treated cases; however, microcystic lesions were also represented, indicating that lesion morphology alone may not be sufficient to determine postnatal surgical requirement. Overall, our findings support current evidence indicating that surgical decision-making in CPAM should be guided not by prenatal diagnosis alone, but by the presence of prenatal risk markers and postnatal symptomatology^14^.

Although some centers advocate prophylactic surgical resection due to potential risks of malignancy and infection^14^, our findings—demonstrating that only 12% of live-born infants required surgery—support the safety and effectiveness of a “watchful waiting” strategy in most cases. The low rate of postnatal surgery and the absence of CPAM-related mortality in our cohort further strengthen the argument for avoiding invasive interventions in asymptomatic, low-risk patients.

Our findings are consistent with previous studies indicating that the majority of fetuses diagnosed prenatally with CPAM, particularly those with low-risk prenatal characteristics, have favorable perinatal and long-term outcomes. Overall, our results reinforce the prognostic value of CVR and support a conservative management strategy in asymptomatic, low-risk fetuses.

### Limitations

The retrospective and single-center design of this study represents its main limitation. Additionally, the absence of routine postnatal computed tomography in all cases limits complete radiological documentation of lesion regression. Nevertheless, the inclusion of a homogeneous cohort of 56 cases and a relatively long mean follow-up period of 72.5 months constitutes a major strength of this study, providing valuable insights into the postnatal natural history and long-term outcomes of CPAM.

## CONCLUSION

In conclusion, the prognosis of fetuses diagnosed prenatally with CPAM is generally favorable. CVR remains the most important measurement for predicting fetal hydrops and adverse perinatal outcomes. Our findings strongly support conservative postnatal management as the first-line approach in asymptomatic, low-risk cases, given the high rate of spontaneous regression and low requirement for surgical intervention.

## Data Availability

The datasets generated and/or analyzed during the current study are available from the corresponding author upon reasonable request.
